# Samuel Thomas von Sömmerring’s Contributions on the Cranial Nerves and Vomeronasal Organ

**DOI:** 10.7759/cureus.2859

**Published:** 2018-06-22

**Authors:** George S Stoyanov, Boyko K Matev

**Affiliations:** 1 General and Clinical Pathology/Forensic Medicine and Deontology, Medical University, Varna, BGR; 2 Medicine, Medical University, Varna, BGR

**Keywords:** samuel thomas von sömmerring, vomeronasal organ, cranial nerves, jacobsons organ, history of anatomy, olfaction, pheromones, pioneer, pterodactyl, cranial nerves classification

## Abstract

Samuel Thomas von Sömmerring (January 28^th^, 1755, Thorn, then Royal Prussia, now Torun Poland – March 2^nd^, 1830, Frankfurt am Main, then a free city, now Germany) was one of the most respected Germanic scientists of his time. Whilst working on his philosophy doctorate (Ph.D.) thesis, when he was only 23 years old (circa 1778), Sömmerring proposed a new classification for the arrangement of the cranial nerves, based on the order in which they become visible on the surface of the brain. Amongst his many other anatomical studies worthy of notice, in 1809 Sömmerring began studying the human olfactory system. During this period, he published a detailed text with sketches, being the first to describe in detail the human vomeronasal organ (VNO), working in parallel with Jacobsen, whose name has been synonymous with the VNO, despite denying its existence in man. Nonetheless, Sömmerring's contributions are numerous. Some of his other works include the description of the structure of the female skeleton and how it differs from the male and the first description of the Pterodactyl in 1812, with which he has been epitomized in modern times and denoted due to his erroneous concepts on it. Even though he studied a wide range of subjects from medical to political, most of his work has been overlooked or forgotten but it is important to understand the range of his contributions.

## Editorial

**Figure 1:**
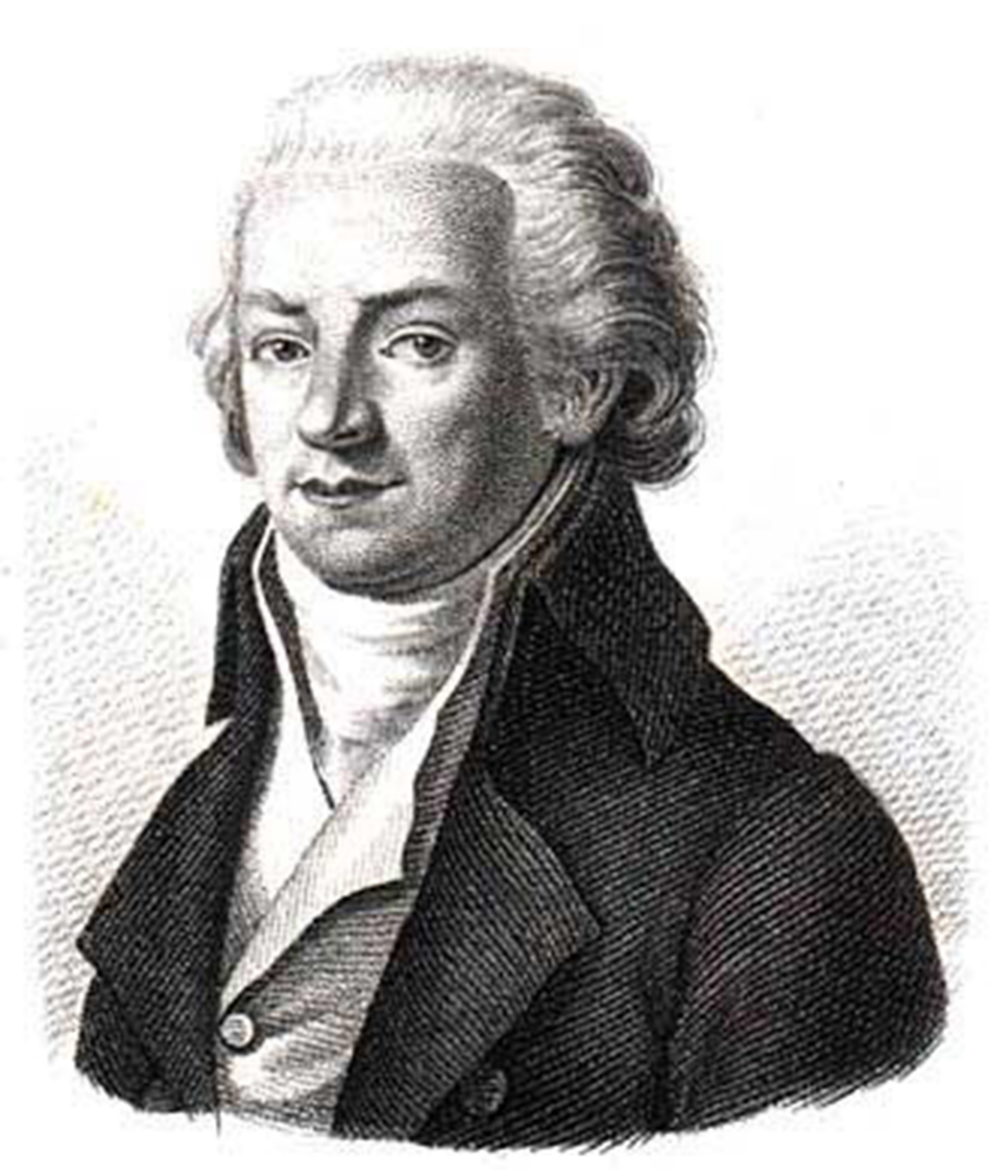
Samuel Thomas von Sömmerring

Samuel Thomas von Sömmerring (January 28^th^, 1755, Thorn, then Royal Prussia, now Torun Poland – March 2^nd^, 1830, Frankfurt am Main, then a free city, now Germany) was one of the most respected Germanic scientist of his time (Figure 1) [1].

He completed his school education in his hometown in 1774 and enrolled in medicine in the University of Göttingen in the same year. Immediately after graduation, he became an assistant professor in Anatomy at the Collegium Carolinum and later at the University of Mainz.

Whilst working on his philosophy doctorate (Ph.D.) thesis, when he was only 23 years old (circa 1778), Sömmerring proposed a new classification for the arrangement of the cranial nerves, based on the order in which they become visible on the surface of the brain. This expanded and revised on the previous classifications introduced from Galen and Vesalius, whose works contained only seven cranial nerves and Willis’s containing ten [2]. This classification is still widely used today, despite two new cranial nerves being described since then, the terminal (first described in 1870 in sharks and in 1914 in humans) and intermediate (first described in 1563, but still widely regarded as part of the facial nerve) nerves, due to its simplicity and compact arrangement [3]. This work also included the connection of the optical nerve to the retina and macula, which Sömmerring was also the first to describe.

Amongst his many other anatomical studies, in 1809 Sömmerring began studying the human olfactory system. During this period, he published a detailed text with sketches, being the first to describe in detail the human vomeronasal organ (VNO), working in parallel with Jacobsen [4]. Unfortunately since then the text and sketches have been lost, Sömmerring‘s contributions to the discovery of the human VNO have since been widely forgotten. Nonetheless, we can see the impact the text had on his contemporaries, who highly praise Sömmerring’s findings and statements. The following statements on Sömmerring’s influential contributions on the topic can be found in M. Potiquet’s masterpiece Le canal de Jacobson. De la possibilité de le reconnaitre sur le vivant et de son role probable dans la pathogénie de certaines lésions de la cloison nasale (Of Jacobson’s Canal. Of the Possibility of Locating it in Living Beings and of its Possible Role) [4-5]:

Noticed and described in humans by Fr. Ruysch and later by S. Th. Sömmerring, in his magnificent graphics on the anatomy of the olfactory organ, this small canal has been, since the discovery of Jacobson’s organ in mammals (1811), mentioned in humans by J. Fr. Merkel and studied in the human embryo by Dursy.

Its existence in man is constant, claims Sömmerring. We would not be as affirming, at least in regards to adults or the elderly.

He describes it surrounded by a bead, he is correct; however, in adolescents and in adults, if we believe the anatomic pieced that we have had before our eyes and our findings in the living, if we have faith in the figures of the septum in which it is found (Sömmerring, A. Köelliker, Schwalbe, Merkel), this orifice presents itself mostly limited by a valvula; the bead does not seem to exist at all but the youngest of children (See the figure in Ruysch's work), and if M. Moldenhauer did not manage to locate it in the living, it is probably because he had begun looking for a bead as a landmark.

Taking into regard the works of this great anatomist on the cranial nerves and VNO, perhaps if the terminal nerve had been discovered earlier, its connection to the VNO may have been described by Sömmerring, giving it, greater impact and his Ph.D. thesis would have proposed a different classification of the cranial nerves.

Sommerring made numerous anatomic contributions. Some of his other works include the description of the structure of the female skeleton and how it differs from the male and the first description of the Pterodactyl in 1812. Sommerring made numerous observations and contributions across wide-ranging areas from medicine, archeology, paleontology, refining the telescope and telegraph to politics and it is unfortunate that many of the works were lost and an interesting observer such as this has been mostly forgotten.
